# Polyphenol-Hydroxylating Tyrosinase Activity under Acidic pH Enables Efficient Synthesis of Plant Catechols and Gallols

**DOI:** 10.3390/microorganisms9091866

**Published:** 2021-09-02

**Authors:** Hanbit Song, Pyung-Gang Lee, Hyun Kim, Uk-Jae Lee, Sang-Hyuk Lee, Joonwon Kim, Byung-Gee Kim

**Affiliations:** 1 School of Chemical and Biological Engineering, Seoul National University, Seoul 08826, Korea; kevin7933@snu.ac.kr (H.S.); lpg2314@snu.ac.kr (P.-G.L.); dotheg7@snu.ac.kr (H.K.); ujlee@snu.ac.kr (U.-J.L.); sanglee81@gmail.com (S.-H.L.); kjw1024@snu.ac.kr (J.K.); 2 Institute of Molecular Biology and Genetics, Seoul National University, Seoul 08826, Korea; 3 Institute of Engineering Research, Seoul National University, Seoul 08826, Korea; 4 Bio-MAX/N-Bio Institute, Seoul National University, Seoul 08826, Korea; 5 Institute for Sustainable Development (ISD), Seoul National University, Seoul 08826, Korea

**Keywords:** plant polyphenols, flavonoids, monooxygenases, tyrosinases, *ortho*-hydroxylation, biotransformation

## Abstract

Tyrosinase is generally known as a melanin-forming enzyme, facilitating monooxygenation of phenols, oxidation of catechols into quinones, and finally generating biological melanin. As a homologous form of tyrosinase in plants, plant polyphenol oxidases perform the same oxidation reactions specifically toward plant polyphenols. Recent studies reported synthetic strategies for large scale preparation of hydroxylated plant polyphenols, using bacterial tyrosinases rather than plant polyphenol oxidase or other monooxygenases, by leveraging its robust monophenolase activity and broad substrate specificity. Herein, we report a novel synthesis of functional plant polyphenols, especially quercetin and myricetin from kaempferol, using screened bacterial tyrosinases. The critical bottleneck of the biocatalysis was identified as instability of the catechol and gallol under neutral and basic conditions. To overcome such instability of the products, the tyrosinase reaction proceeded under acidic conditions. Under mild acidic conditions supplemented with reducing agents, a bacterial tyrosinase from *Bacillus megaterium* (*Bm*Ty) displayed efficient consecutive two-step monophenolase activities producing quercetin and myricetin from kaempferol. Furthermore, the broad substrate specificity of *Bm*Ty toward diverse polyphenols enabled us to achieve the first biosynthesis of tricetin and 3′-hydroxyeriodictyol from apigenin and naringenin, respectively. These results suggest that microbial tyrosinase is a useful biocatalyst to prepare plant polyphenolic catechols and gallols with high productivity, which were hardly achieved by using other monooxygenases such as cytochrome P450s.

## 1. Introduction

Tyrosinase is a di-copper containing oxygenase that catalyzes phenolic compounds into catechols, and subsequent its catecholase activity converts the catechols into the corresponding quinones, finally leading to the synthesis of biological melanins [1,2,3]. The widespread presence of tyrosinases across bacteria, animals, plants, and fungi enables us to generate a vast library of the oxygenases characterized by broad spectra of molecular structures, substrate specificities, and catalytic properties. Above all, tyrosinase hydroxylates L-tyrosine (i.e., monophenolic substrate) at its *ortho*-position of its hydroxyl group and further oxidizes the formed di-hydroxy catechol compound, i.e., L-3,4-dihydroxyphenylalanine(L-DOPA), into the corresponding quinone compound. The enzyme usually has conserved di-Cu-coordinating six histidine residues as their essential structural homology sequences. Among its various applications, phenol detection/treatment in wastewater and melanin pigment synthesis are worth to noting as major ones. Recently, it was reported that some tyrosinases could become efficient biocatalysts to prepare large scale *ortho*-hydroxylated plant polyphenols [4,5,6]. In the biotransformation, monophenolase activity was selectively used for *ortho*-hydroxylation of daidzein, genistein, and resveratrol, while diphenolase (catecholase) activity was suppressed by catechol chelation by borate buffer and excess usage of antioxidants such as ascorbic acid. For diphenolase activity, melanins or melanin-like compounds are final products that can be applied for MRI reagents [7], photo-thermal/acoustic materials [8,9], or even structural coloring materials [10]. Moreover, the quinolic intermediate generated through diphenolase activity can form hydrogels from proteinous polymers (e.g., collagen, gelatin) and tyramine-modified carbohydrate polymers (e.g., hyaluronic acid).

Plant polyphenols are biological, functional, secondary metabolites composed of multiple phenolic rings. For example, quercetin and myricetin are kaempferol-derived flavonols that share a hydroxyl group at C3 and have additional catechol or gallol moieties at their B-ring, respectively. The roles of polyphenols in the plant include defending themselves from herbivores through bitter tastes, bridging a chemical communication with symbiotic microbes as transcriptional regulators having binding affinity to its corresponding receptors, and generating flower coloration [11,12,13]. Apart from such natural roles, polyphenols also display various supplementary dietary effects such as antioxidative, antimicrobial, or anticancer activities. For example, myricetin is one of the most important polyphenols categorized as a flavonol, giving diverse biological benefits such as anticancer, antiviral, antithrombotic, anti-atherosclerotic, and antidiabetic functions. In a clinical study, relatively low incidence rates of several chronic diseases were significantly associated with high intake of myricetin as well as other flavonoids comprising quercetin, kaempferol, naringenin, and hesperetin. Additionally, a recent study found that myricetin is effective in treating the infection of the Ebola virus [14]. Due to such medicinal and clinical values of these polyphenols, preparations of polyphenolic substances such as various nutraceuticals or drugs have been attempted for a long time, using solvent extraction from natural plant resources [15,16,17]. However, solvent extraction has issues on labor-intensive gathering natural resources, usage of a large amounts of non-ecofriendly organic solvents and irregularity of bio-active ingredients depending on the harvest conditions of sources [18,19]. Biosynthesis of polyphenolic compounds in the industry might be an overcoming solution to these issues. Recently, quite remarkable but partial successes have been made in the production of flavonoids, isoflavonoids, stilbenoids, and their derivatives using either microbial biotransformations or microbial production systems with metabolic engineering [20,21,22,23,24].

Still, some polyphenolic derivatives can hardly be prepared for practical commercial usage. One of the most challenging compounds is myricetin which naturally comes from the biosynthetic pathway of flavonols, where a plant cytochrome P450 (CYP), CYP75A, known as flavonoid 3′,5′-hydroxylase (F3′5′H), conducts two consecutive hydroxylation at C-3′ and 5′ of kaempferol, converting it into myricetin [25]. Recently, a study reported that a *Saccharomyces cerevisiae* strain comprised of plant-derived flavonoid 3-hydroxylase (F3H), flavonoid 3′-hydroxylase (F3′H), F3′5′H, and flavonol synthase (FLS) succeeded to produce 145 mg/L of myricetin from naringenin after 3 days of cultivation [21]. However, these P450s are NAD(P)-dependent, so they only work in vivo or with the supplement of high concentration of NAD(P). Furthermore, their poor functional expression and low catalytic activities of the CYP enzymes in heterologous microbial hosts gave poor bioconversion yields. Except for P450s, there have been no other alternative biocatalysts synthesizing such kinds of gallol flavonoids.

**Figure 1:**
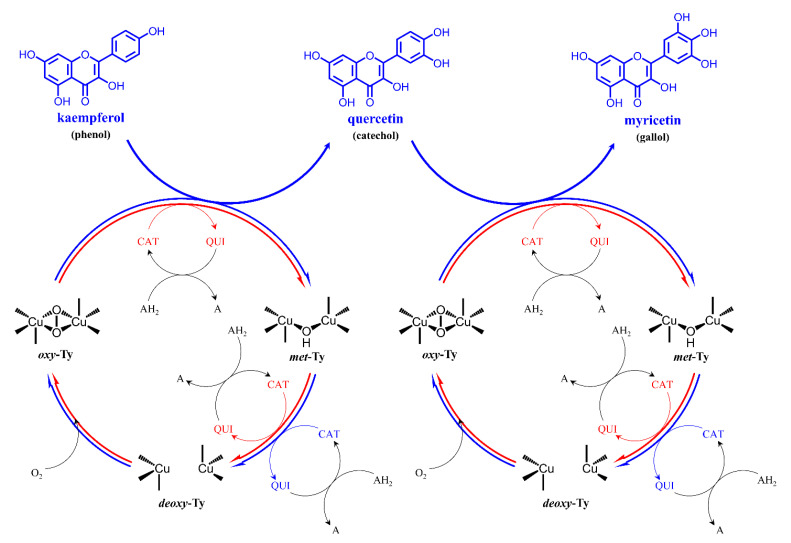
Schematic representation of plant polyphenol oxidation in the general tyrosinase mechanism. The two main cycles of tyrosinase, monophenolase cycle and diphenolase cycle, were represented with blue and red arrows, respectively. Some chemical species are described with abbreviations: those are: A: L-dehydroascorbic acid, AH_2_: L-ascorbic acid, Ty: tyrosinase, CAT: catechol, GAL: gallol, QUI: *o*-quinone.

Herein, a remarkable success in the synthesis of catechol and subsequent gallol from kaempferol is reported using a bacterial tyrosinase (Figure 1). In the efficient synthetic processes for such valuable polyphenolic products, the high instability of the plant polyphenols under the tyrosinase reaction condition was a primary obstacle. To overcome such problems, the biotransformation was attempted under the mild acidic condition of pH 6.0, considering the optimal pH of the tyrosinase reaction (ca. pH 8.0) and the auto-oxidation of myricetin or quercetin often induced under alkaline conditions. Thus, kaempferol was converted into quercetin with a yield of 60% equivalent moles of initial kaempferol, and myricetin production was also possible, up to 57 μM (ca. 6% yield and 18 mg/L/h productivity for 1 mM kaempferol) with consecutive two-step oxidative hydroxylations. In addition, the synthesis of catechol and gallol derivatives from apigenin or naringenin was confirmed with MS identification, which implied a broad substrate window of the bacterial tyrosinase.

## 2. Materials and Methods

### 2.1. Materials

Kaempferol, quercetin dehydrate, and myricetin were purchased from Toronto research chemicals (Toronto, ON, Canada), Riedel-de Haën (Honeywell, Charlotte, NC, USA), and Fluka (Honeywell, Charlotte, NC, USA), respectively. The other flavonoids, apigenin, and *rac*-naringenin and the other compounds were purchased from Sigma Aldrich (Merck KGaA, Darmstadt, Germany). All flavonoids were dissolved in specific amounts of dimethyl sulfoxide (DMSO) with 50 or 100 mM as stock concentrations. As microbial sources for cloning of *Kg*Ty and *Pg*Ty, *Kitasatospora griseola* MF730-N6 and *Photobacterium galatheae* S2753 were kindly provided by Russell G. Kerr (University of Prince Edward Island, Charlottetown, PE, Canada) and Lone Gram (Technical University of Denmark, Kongens Lyngby, Denmark), respectively. *Burkholderia cepacia* KCCM 41422 (ATCC 25416) for cloning of *Bc*Ty was obtained from the Korean Culture Center of Microorganisms.

### 2.2. Measurement of Flavonoid pH Stability

In the sample volume of 400 μL, 500 μM of final quercetin or myricetin concentration was dissolved in the 50 mM buffer of pH 5.0~8.0 containing 1% (*w*/*v*) polyvinylpyrrolidone (PVP), and the samples were incubated at 37 °C with gentle shaking (200 rpm). Citrate-phosphate buffer (pH 5.0~7.0) and Tris-HCl buffer (pH 8.0) were used for the buffering system and PVP was added to dissolve flavonoids [26]. Sampling time points were at 1, 2, 6, and 24 h after the incubation started. For quercetin, 40 μL of aliquots were five times diluted with 160 μL of HPLC grade methanol and these diluted samples were analyzed by HPLC. For myricetin, 50 μL of aliquots were diluted four times with 150 μL of DW, and extracted with 1 mL of ethyl acetate (EA). EA part of 500 or 300 μL was retrieved and vacuum evaporated. These evaporated samples were dissolved with 100 μL of HPLC grade methanol and analyzed by HPLC.

### 2.3. Tyrosinase Activity Assay

Enzyme expression and purification were conducted with slight modifications of previous work [27]. Six bacterial tyrosinases had been transformed in *E. coli* BL21 (DE3) and their cell stocks were used to express each tyrosinase (Table S1). Each cell was inoculated in 1.1 mL of Luria-Bertani lysogeny broth with the desired antibiotic (LB) depend on each vector and cultured under 37 °C with gentle shaking (200 rpm) for 8 h. Each 0.2 mL cultured inoculum was added to 10 mL of LB and incubated under 37 °C with gentle shaking until the turbidity (OD_600_) became 0.8~1.1. At the end of incubation, isopropyl β-D-1-thiogalactopyranoside (IPTG) induction was conducted in the incubator of 18 °C, 200 rpm for overnight (ca. 18 h) with the addition of 0.5 mM CuSO_4_ and 0.2 mM IPTG as final concentrations. After IPTG induction, each cell was collected and washed with phosphate-buffered saline (PBS) using a refrigerated centrifuge operated at 4000 rpm and 4 °C. The washed cells were resuspended in 1 mL of 50 mM Tris-HCl buffer pH 8.0 with 0.25 mM phenylmethylsulfonyl fluoride (PMSF). The cell suspensions were sonicated and centrifuged at 16,000 rpm, 4 °C. The supernatants were injected in columns packed with Ni-NTA agarose bead (Qiagen Korea Ltd., Seoul, Korea) and the 6x His-tagged tyrosinases were purified. The purified tyrosinases were dialyzed by ultrafiltration with Amicon Ultra-15 (Merck Millipore, Ltd., Seoul, Korea).

To evaluate the activities of tyrosinases against kaempferol or quercetin at acidic pH range of 4.0~6.0, a colorimetric assay was exploited with the aid of MBTH. In each well of 96-well plate, 200 μL of 50 mM buffer (pH 4.0: citrate buffer, pH 5.0, 6.0: citrate-phosphate buffer) solution mixture containing 10 μM CuSO_4_, 5 mM MBTH, 0.5 μM tyrosinase, 1% (*w*/*v*) PVP and 0.5 mM kaempferol or quercetin was examined. For the blank control, 50 mM, pH 8.0 Tris-HCl buffer was added instead of tyrosinase. These 200 μL mixtures were prepared by mixing two parts: 50 μL of 4× substrate mixture containing 4% (*w*/*v*) PVP 40k and 2 mM kaempferol or quercetin and 150 μL of tyrosinase mixture, which contained the others to complete the mixture. These two parts were incubated under 37 °C separately for 10 min and mixed. Soon after, absorbance at 505 nm of each well was measured for an hour, with an interval of 30 s, using a UV/Vis spectrophotometer (SPECTROstar Nano; BMG Labtech GmbH, Ortenberg, Germany). In addition, by measuring the absorbance at 475 nm, which is the maximal absorption wavelength of dopachrome, activities on L-tyrosine and L-DOPA of *Bm*Ty were obtained under pH range of 5.0~8.0 [28]. For the dopachrome assay, in each well of 96-well plates, 200 μL of 50 mM buffer (pH 5.0~7.0: citrate-phosphate buffer, pH 8.0: Tris-HCl buffer) solution mixture containing 10 μM CuSO_4_, 0.1 μM *Bm*Ty, and 1 mM L-tyrosine was used. Similar to the above procedure, a blank control was prepared. The absorbance at 475 nm was measured for 30 min with an interval of 30 s. The amounts of *Bm*Ty needed for conversion of 1 μmol L-tyrosine (monophenolase activity) or L-DOPA (diphenolase activity) per minute were defined as 1 enzyme unit (U) at different pHs, and the enzyme units of monophenolase activity deduced from this activity test (Table 1) utilized for further experiments.

### 2.4. Kaempferol Conversion Using BmTy at Various pHs

In 1 mL reaction volume, 0.2 mM kaempferol, 1% (*w*/*v*) PVP 40k, 1% (*v*/*v*) DMSO, 3 mM L-ascorbic acid, 2 μM CuSO_4_ and 50 mU/mL *Bm*Ty (427, 216, 133 and 181 nM enzyme at pH 5, 6, 7 and 8, respectively, Table 1) were added into 50 mM buffer (pH 5.0~7.0: citrate-phosphate buffer, pH 8.0: Tris-HCl buffer) as final concentrations. Aliquots of 100 μL were vortexed with 400 μL EA at specific sampling points in time, and 200 μL of EA parts were retrieved and dried. The residues were dissolved with 100 μL MeOH and analyzed with HPLC.

### 2.5. MS Identification of Various Ortho-Hydroxylated Products

Three polyphenol substrates, kaempferol, apigenin, *rac*-naringenin, were used to assess the hydroxylation ability of *Bm*Ty on broad substrates. For kaempferol, 500 μL of reaction mixture containing 0.2 mM kaempferol, 3% (*w*/*v*) PVP 40k, 25 mM L-ascorbic acid (LAA), 10 μM CuSO_4_, 1 μM *Bm*Ty and 50 mM citrate-phosphate buffer pH 6.0 was incubated at 37 °C with shaking (200 rpm) for 2 h. For apigenin, all the other reaction condition were the same as kaempferol sample, but 0.2 mM apigenin, instead of kaempferol, and 5% (*w*/*v*) PVP 40k were used. For *rac*-naringenin, 400 μL of reaction mixture containing 1 mM *rac*-naringenin, 5% (*w*/*v*) PVP 40k, 25 mM L-ascorbic acid, 10 μM CuSO_4_, 1 μM *Bm*Ty and 50 mM citrate-phosphate buffer pH 6.0 was incubated at 37 °C with shaking (200 rpm) for 3 h. In the case of kaempferol and apigenin samples, 400 μL of aliquot was retrieved and diluted with 1200 μL of DW. Each diluted sample was extracted with 2 mL of EA and 1.6 mL of EA was retrieved and vacuum-evaporated. For the *rac*-naringenin reaction mixture, 50 μL of aliquot was retrieved and diluted with 150 μL of DW. This diluted sample was extracted with 1 mL of EA and 0.8 mL of EA was retrieved and vacuum-evaporated. The evaporated samples from kaempferol or *rac*-naringenin reactions were dissolved in 100 μL of EA and added 2 μL of *N*,*O*-Bis(trimethylsilyl)trifluoroacetamide (BSTFA) to derivatize trimethylsilyl (TMS) group. The TMS derivatization was conducted under 37 °C for 20 min. The 0.5 or 1 μM of authentic references were also dissolved in EA and these authentic samples were TMS-derivatized, as with other analyzed samples. These derivatized samples were analyzed with GC-MS. Evaporated residue from apigenin reaction was dissolved in 200 μL of methanol and filtered with 3 kDa cut-off centrifugal filter (Merck). The flow-through was analyzed with HPLC-ESI-MS/MS.

For GC-MS analysis, TRACE GC Ultra gas chromatograph (GC) coupled with ITQ1100 ion trap mass detector (Thermo) was used. In GC, a nonpolar capillary column (5% phenyl methyl siloxane capillary 30 m × 250 μm i.d., 0.25-μm film thickness, TR-5 ms) was equipped. Each 1 μL sample was injected with the splitless mode of 0.8 min splitless time. The oven schedule started with the initial temperature of 65 °C and held for one minute, and then the oven temperature increased to 250 °C, with the ramp rate of 30 °C/min. After 3 min of hold time at 250 °C, the second ramp proceeded with the rate of 5 °C/min until the temperature reached 300 °C. Finally, the temperature was held for 20 min at 300 °C. The ion source temperature, flow rate of carrier gas (Helium), and electron energy of EI were 230 °C, 1 mL/min, and 70 eV, respectively.

For HPLC-ESI-MS/MS analysis, TSQ Quantum Access Max coupled with Accela HPLC system (Thermo) equipped with the COMOSIL 5C_18_-AR-II column (5 μm particle size, 4.6 mm I.D. × 150 mm; Nacalai Tesque, Kyoto, Japan) was used. In HPLC, eluent of 65% water with 0.1% formic acid and 35% acetonitrile with 0.1% formic acid passed through the C_18_ column with the flow rate of 0.4 mL/min. Each 10 μL sample was injected into HPLC and analyzed with ESI-MS/MS. The condition for mass spectrometry was as follows: spray voltage, 3.0 kV; vaporizer temperature, 573 K; capillary temperature, 593 K; sheath gas pressure, 30 psi; aux gas pressure, 10 psi. ESI-MS/MS was operated in positive ion mode and two scan events occurred per acquired ions. The first scan event was a full scan in the mass range of *m*/*z*: 200~600. The second scan was a dependent scan that the most intense ion in the first scan was fragmented with the collision energy of 35 eV and scanned.

### 2.6. Biosynthesis of Quercetin and Myricetin Using BmTy

As a reaction mixture solution, 400 μL of 50 mM citrate-phosphate buffer (pH 6.0) solution containing 1 μM *Bm*Ty, 10 μM CuSO_4_, 3% (*w*/*v*) PVP 40 k, 25 mM LAA, and 0.2~2 mM kaempferol were used. DMSO was adjusted to make 4% (*v*/*v*) of a final DMSO concentration for all the samples. One-hour reaction was performed in an orbital shaker with 200 rpm at 37 °C. The reaction was finished by EA extraction. For the sample extraction, 50 μL aliquot of the reaction mixture was mixed with 150 μL DW and then extracted with 1 mL EA. EA part of 800 μL was retrieved and vacuum evaporated. This dried sample was dissolved with 100 μL of HPLC-grade methanol and analyzed with HPLC.

### 2.7. HPLC Analysis

For HPLC analysis, HPLC (YL9100 HPLC; YoungLin, Anyang, Korea) equipped with the COMOSIL 5C_18_-AR-II column was used. Each 10 μL sample was injected and the flow rate was 0.7 mL/min. The elution ratio of water with 0.1% (*v*/*v*) trifluoroacetic acid to acetonitrile was 65:35 (volume ratio). The flow-through from the C_18_ column was measured by UV detector at the wavelength of 376 nm. The calibration curves of flavonoids were obtained by measuring 10, 100, 500, and 1000 μM samples dissolved in HPLC grade methanol. By comparing these calibration curves, the concentration of each sample was measured for quantification analysis.

### 2.8. Preliminary Enzyme Deactivation Tests with the Incubation of Flavonols

In each well of 96-well plates, 280 μL of 50 mM citrate-phosphate buffer (pH 6.0) solution containing final concentrations of 2 μM CuSO_4_, 0.5 mM kaempferol, quercetin or myricetin, 0.5% (*v*/*v*) DMSO, 4% (*w*/*v*) PVP 40 k, and 0.5 μM *Bm*Ty was added. Flavonols were omitted for negative control and 0.5 mM L-tyrosine was added instead of flavonols for positive control. For blank, flavonols, *Bm*Ty, and L-tyrosine were all omitted (Figure 8A). For Figure 8B, almost the same components were added except final concentrations of 0.1~0.4 mM kaempferol, quercetin or myricetin, 0.8% (*v*/*v*) DMSO and 0.4 μM *Bm*Ty were used, instead. They were all incubated at 37 °C, and at specific incubation points in time, the incubated solutions were diluted five times (for Figure 8A) or ten times (for Figure 8B) in the buffers containing 50 mM, pH 8.0 Tris-HCl buffer, and 1 mM L-tyrosine as final concentrations and the tyrosinase reaction was started. The reactions were proceeded at 37 °C and monitored at the 475 nm wavelength for dopachrome assay, and initial rates were calculated. The initial rates were treated as residual initial rates (*V*_res_) at specific incubation times (*t*_i_) and regressed using the following equation to obtain the ] deactivation coefficients (*k*_d_), with an assumption such that the concentration of L-tyrosine is far above *K*_M,_ such that the initial rate of the native enzyme is identical to *V*_max_.

$$\ln\left(\frac{V_{\mathrm{res}}}{V_{0}}\right)=-k_{d}t_{i}$$

where *V*_0_ is the residual initial rate at *t*_i_ = 0. Based on the following chemical equations and assuming that the concentration of an incubated deactivator [D] is constant during incubation (because of its high concentration compared to [E]),

$$E\overset{k_{d1}}{\to }E_{d},\;E+D\overset{k_{d2}}{\to }E_{d}$$

*k*_d_ can be more precisely expressed with the equation shown below.

$$k_{d}=k_{d1}+k_{d2} [D]$$

*k*_d1_ is spontaneous deactivation constant of the enzyme and *k*_d2_ is suicide deactivation constant of the enzyme by a deactivator. Therefore, if a suspected deactivator deactivates the enzyme in a suicidal manner, the *k*_d_ value will be changed linearly according to [D].

### 2.9. BmTy Inhibition Kinetics of Quercetin

In 96-well plate, each well of 200 μL aqueous solution was added to make a final solution of 50 mM of Tris-HCl buffer pH 8.0, 10 μM of CuSO4, 0~100 μM of quercetin, 0.1% (*v*/*v*) of DMSO, 25~100 μM of L-tyrosine and 0.1 μM of *Bm*Ty. Michaelis–Menten kinetics were measured by monitoring 475 nm dopachrome wavelength and recording the initial rate by varying quercetin and L-tyrosine concentrations. Each sample was triplicated, and the data were shown as averages with standard errors. The apparent *k*_cat_ ($k_{\mathrm{cat}}^{\mathrm{app}}$) and *K*_M_ ($K_{M}^{\mathrm{app}}$) values were obtained by non-linear regression using the following equation.

$$V_{i}=\frac{k_{\mathrm{cat}}^{\mathrm{app}}{ [E]}_{t}{\left[S\right]}_{i}}{K_{M}^{\mathrm{app}}+{ [S]}_{i}}$$

*V*_i_, is the initial reaction rate, and [E]_t_ and [S]_i_ are initial *Bm*Ty concentration and initial L-tyrosine concentration, respectively. Inhibition constants were obtained from two apparent constants by regressing the following equations.

$$\frac{K_{M}^{\mathrm{app}}}{K_{M}}\frac{k_{\mathrm{cat}}}{k_{\mathrm{cat}}^{\mathrm{app}}}=1+\frac{ [I]}{K_{I1}},\;\frac{k_{\mathrm{cat}}}{k_{\mathrm{cat}}^{\mathrm{app}}}=1+\frac{ [I]}{K_{I2}}$$

[I], *K*_I1_ and *K*_I2_ are quercetin concentration, competitive inhibition constant, and uncompetitive inhibition constant, respectively.

## 3. Results

### 3.1. Measurement of pH Stability of Quercetin and Myricetin

**Figure 2:**
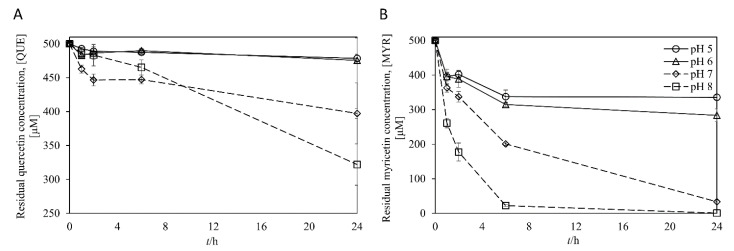
pH stability of quercetin (**A**) and myricetin (**B**). 500 μM of the polyphenols were incubated under respective pH at 37 °C. Each data point was indicated as mean values with standard deviations (error bars) from three independent replicates.

In flavonoids oxidation reaction such as biosynthesis of *ortho*-hydroxylated plant polyphenols (i.e., quercetin and myricetin) using tyrosinase, it was well known to a certain extent that auto-oxidation of *ortho*-hydroxylated polyphenols at alkaline pH conditions would be the main problem [29,30,31,32,33]. Therefore, we first measured the stability of the quercetin and myricetin as representatives of catechol and gallol compounds, respectively, within the pH range of 5 to 8. Although kaempferol was stable in the neutral aqueous solution of up to pH 7.0 for 4 days [34], quercetin and myricetin were susceptible to oxidation particularly under alkaline-to-neutral conditions (Figure 2). Quercetin was stable in the moderately acidic condition (ca. pH 5 to 6), while it was rapidly oxidized at pH 7 to 8 (i.e., 40% of quercetin was oxidized after 24 h at pH 8). Moreover, myricetin was more vulnerable at neutral or basic pH than quercetin. At above pH 7, most of the myricetin was oxidized within 24 h (Figure 2). The results imply that autooxidation of quercetin and myricetin at neutral and basic pH is attributed to alkali-sensitive catechol and gallol moieties, respectively, which are unique functional groups of those flavonols [35,36]. As a result, the acidic condition is desirable for efficient synthesis of the catechol and gallol, also suggesting that potentially good flavonol oxygenases should exhibit significant catalytic activity under the acidic pH (i.e., ≤6).

### 3.2. Screening Bacterial Tyrosinases for Polyphenol Conversion under Acidic Condition

Our group has searched various tyrosinases from diversified resources for specialized applications. One of them is a tyrosinase from *Streptomyces avermitilis* (MelC2, and here so-called, *Sa*Ty). This tyrosinase was reported to have substrate specificity not only on some small molecules like L-tyrosine or *trans*-resveratrol [5,37] but also on bio-macromolecules like gelatin or tyramine-modified polysaccharides, such as chitosan and hyaluronic acid [38]. Other tyrosinases from *Kitasatospora griseola* (*Kg*Ty) and *Photobacterium galatheae* (*Pg*Ty) were selected because they are the two representatives of a few subgroups in the cladogram of bacterial tyrosinases built with the aid of Subgrouping Automata program [39].

Most tyrosinases have their optimal activity in neutral-to-slightly alkaline pH (ca. 7~8). Recently, a tyrosinase from *Burkholderia thaliandensis* (*Bt*Ty), which showed potent activity at low pH (ca. pH 4–5) with tetrameric structure, was found [40]. Since *Bt*Ty successfully converted daidzin (isoflavone glycoside), resveratrol (stilbene), and phloretin (chalcone) into their corresponding hydroxylated products, respectively, it was considered as a potential catalyst for the hydroxylation of kaempferol to produce quercetin as well as myricetin. In an effort to discover other acidic tyrosinases, a tyrosinase from *Burkholderia cepacia* (*Bc*Ty), which had high sequence homology with *Bt*Ty, was found.

**Figure 3:**
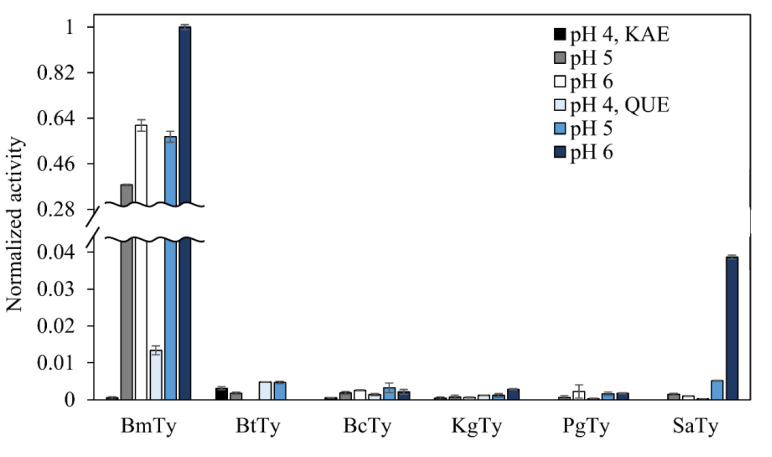
MBTH activity assay for six microbial tyrosinases against kaempferol and quercetin. Each purified tyrosinase (0.5 μM) was measured for its oxidation (or quinone formation) activity against 500 μM of substrates under mild acidic conditions.

Along with the *Bm*Ty which had already been proven to show good activities on various polyphenols [40], the above-mentioned five tyrosinases were assessed for flavonol oxidation screening. However, all the tyrosinases except *Bm*Ty showed marginal activities for kaempferol and quercetin according to MBTH assay (quinone-detecting method) at pH 4 to 6 (Figure 3). In the case of *Bm*Ty, because its optimal pH was around pH 8 [40] the flavonol-hydroxylating activity was relatively higher at slightly acidic pH (i.e., 6) than at acidic pH (i.e., 4 or 5). Since all the rest of the tyrosinases showed marginal activity, *Bm*Ty was chosen to perform further examination.

**Figure 4:**
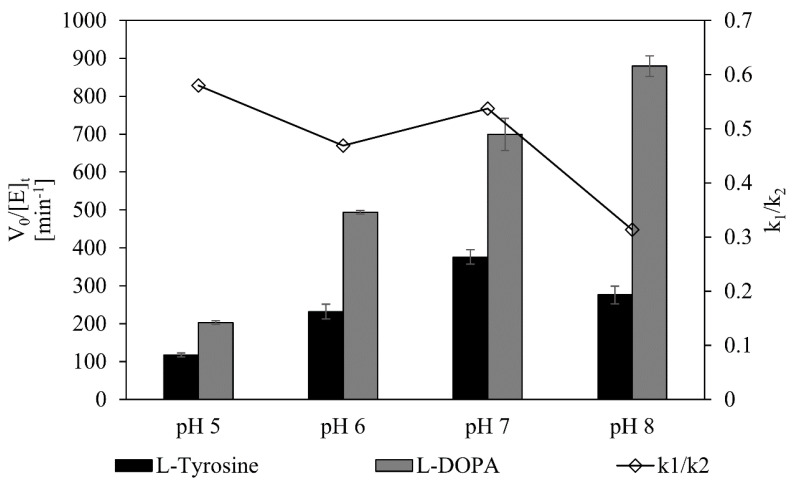
Monophenolase versus diphenolase activity assay of *Bm*Ty in the various pH. 1000 μM of L-tyrosine (black bar) and L-DOPA (gray bar) were used as initial substrates to measure monophenolase (tyrosine hydroxylation activity) and diphenolase activity (quinone formation activity) of *Bm*Ty, respectively.

Next, monophenolase and diphenolase activities of *Bm*Ty were compared in various pHs to examine an optimal condition, where flavonoid hydroxylation (*k*_1_) is favorable, while quinone formation (*k*_2_) is suppressed. For that purpose, the dopachrome assay was conducted using two native substrates of tyrosinases, L-tyrosine and L-DOPA. In general, lowering the reaction pH caused more attenuation of the diphenolase activity (*k*_2_) than the monophenolase activity (*k*_1_), resulting in a higher ratio of monophenolase to diphenolase activity at acidic pH (Figure 4, Table 1). This further suggests that the use of acidic reaction conditions is favorable for flavonol synthesis because diphenolase activity was significantly suppressed, facilitating the accumulation of hydroxylated products.

### 3.3. Effect of pH on the Kaempferol Conversion by BmTy

For the syntheses of hydroxylated compounds, two consecutive monophenolase cycles of tyrosinase were exploited (Figure 1). In the first step where *oxy*-tyrosinase (*oxy*-Ty) took part in the reaction of monophenolase cycle, *ortho*-hydroxylation of the monophenol compound took place and a corresponding catechol compound was generated. To complete a tyrosinase reaction cycle, *met*-tyrosinase (*met*-Ty) subsequently oxidized the just converted catechol compound into the corresponding *o*-quinone compound. *O*-quinones are generated via two routes: one from monophenolase cycle and the other from the two rounds of oxidation steps in the diphenolase cycle. However, the *o*-quinones were reduced to the catechol derivatives by the reducing agent such as L-ascorbic acid (LAA). To be precise, LAA reduces *o*-quinones to catechols by oxidizing itself, becoming L-dehydroascorbic acid, thereby accumulating catechols intact [41].

**Figure 5:**
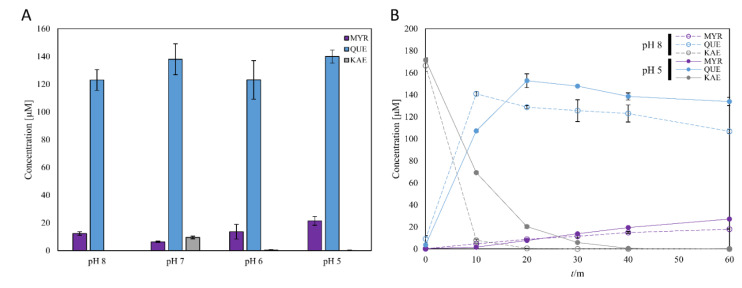
Kaempferol conversion of *Bm*Ty at different pHs. Concentrations of flavonols in one-hour *Bm*Ty reaction at pH 5~8 were shown as gray (kaempferol), blue (quercetin), and purple (myricetin) bars (**A**) and the concentrations of time course reactions at pH 5 and 8 were expressed with the same color for each compound (**B**). Concentrations at pH 8 were expressed with dashed lines and open circles and those at pH 5 were expressed with solid lines and closed circles.

With this reaction scheme, the effect of reaction pH on the reaction rate for the selected tyrosinase was compared using 0.2 mM kaempferol at pH 5~8 with a fixed enzyme unit of 50 mU/mL and 60 min reaction time (Figure 5A). The accumulation of myricetin was generally increased with the decrease in pH. Given this result, we have compared time course reactions at pH 5, where the most produced myricetin was accumulated, and pH 8, where *Bm*Ty showed its optimal activity (Figure 5B). In the reaction time courses using 0.2 mM kaempferol as an initial substrate, the accumulated amount of myricetin at pH 5 exceeded the one at pH 8 in 30 min. Especially, 51% more myricetin was accumulated at pH 5 than that at pH 8 after 60 min of reaction time. Moreover, the concentration of quercetin appeared to be much stabler compound than myricetin at alkaline pH, was higher at pH 5 than at pH 8 after 20 min of reaction time. This result indicates that pH affected the accumulated amount of hydroxylated flavonols within tyrosinase reaction time scale that acidic pH could contribute to the accumulation of the hydroxylated flavonols by preventing auto-oxidation in the buffer. In addition, the higher value of *k*_1_/*k*_2_ ratios at acidic conditions would also be favorable to accumulate the products by avoiding undesirable overoxidation in quinones.

### 3.4. MS Identification of Tyrosinase Products

**Figure 6:**
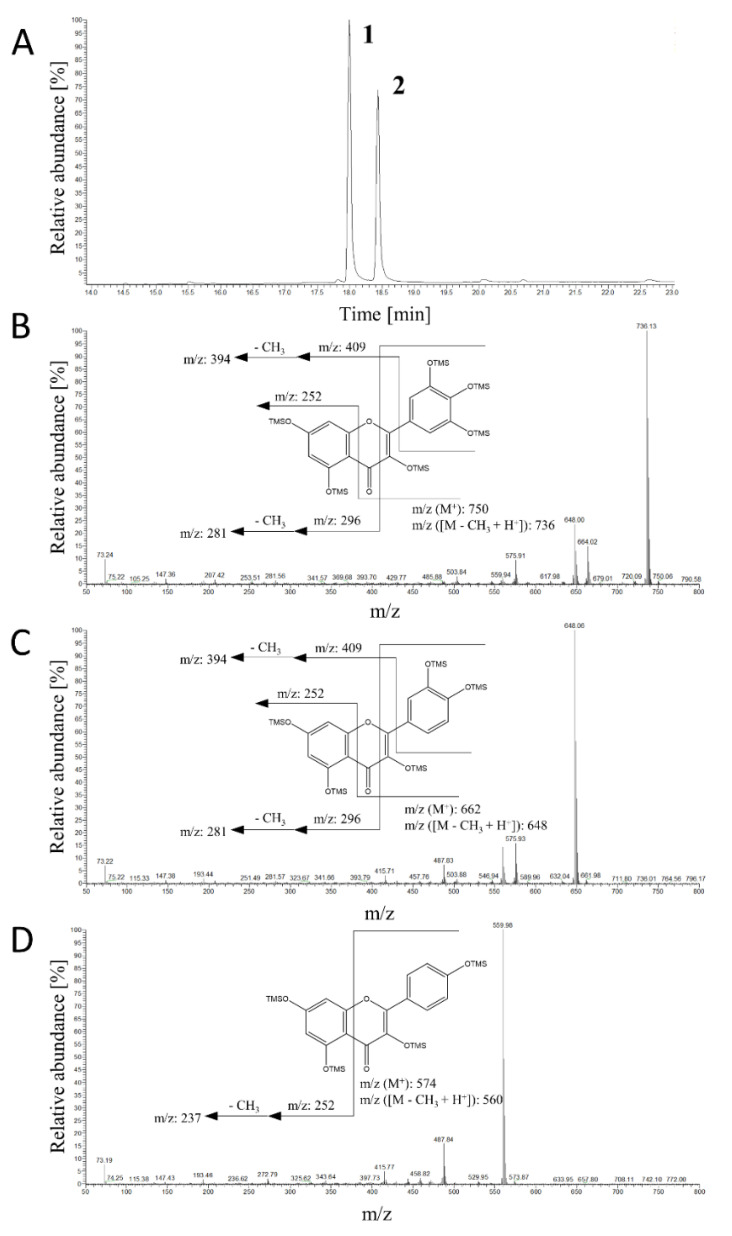
GC/MS analysis for identification of tyrosinase reaction products converted from kaempferol using *Bm*Ty. Two major products at RT = 18.0 min (product 1) and RT= 18.5 min (product 2) were detected, then they were identified as quercetin (*m*/*z* = 662, product 1) and myricetin (*m*/*z* = 750, product 2). (**A**) Gas chromatogram of the reaction sample. (**B**) MS spectrum of the product 2, myricetin. (**C**) MS spectrum of the product 1, quercetin. (**D**) MS spectrum of the standard compound of kaempferol.

Based on the above findings, we carried out *ortho*-hydroxylation of several plant polyphenols namely, kaempferol (flavonol), apigenin (flavone), and *rac*-naringenin (flavanone), using *Bm*Ty below pH 6. According to GC-MS or LC-ESI-MS/MS analysis, *Bm*Ty converted them into their corresponding catechol and gallol derivatives at B-ring (Figure 6, Table S2). For example, kaempferol was efficiently hydroxylated into quercetin (quercetin-5TMS: *m*/*z* = 662, 648, 576, 560), which was subsequently oxidized to myricetin (myricetin-6TMS: *m*/*z* = 750, 736, 664, 648). The EI-mass spectra of these flavonols agreed well with those of authentic chemicals. The overlapping fragmented ions of *m*/*z* = 394 and 281 supported the addition of hydroxyl groups in the B-ring of quercetin and myricetin, respectively. The B-ring-specific hydroxylation was also confirmed in the mass spectra of the reaction products of apigenin and naringenin, indicating luteolin (or tricetin) and eriodictyol (or 3′,5′-dihydroxynaringenin) (Table S2).

### 3.5. Production of Quercetin and Myricetin from Kaempferol Using BmTy

**Figure 7:**
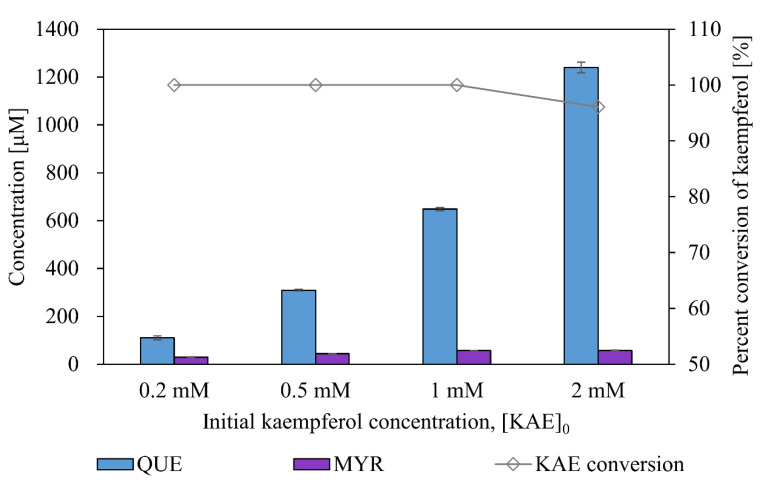
Conversion of kaempferol into quercetin (catechol) and myricetin (gallol) using *Bm*Ty. Purified *Bm*Ty (1 μM) was added to 200~2000 μM of kaempferol and conversion reactions were conducted at pH 6 and 37 °C for 1 h. Concentrations of quercetin (blue bar) and myricetin (purple bar) were measured and substrate conversions were obtained from kaempferol concentrations (line with diamond labels).

To determine if *Bm*Ty can carry out such an efficient B-ring specific hydroxylation, the effect of the initial concentration of kaempferol (0.2–2 mM) on substrate conversion and product concentration were compared (Figure 7). Within the examined concentrations of kaempferol, most of the substrate was converted into quercetin and myricetin in one hour. The titer of quercetin proportionally increased with the initial kaempferol concentration, maintaining a similar yield of ca. 60%. In one-hour reaction, 57 μM of myricetin was produced from 1 mM kaempferol corresponding to 18 mg/L/h productivity. However, myricetin yield did not increase over 57 μM despite using high concentrations of kaempferol loading (ca. 2 mM). Since such flavonols were already known as strong inhibitors against tyrosinase [42,43,44,45], and catecholic or gallol compounds were also known to become suicide deactivators of tyrosinase [46,47,48], the increasing concentrations of the products appeared to inhibit or deactivate *Bm*Ty.

### 3.6. Inhibitory Action of Quercetin in Monophenolase Activity of BmTy

**Figure 8:**
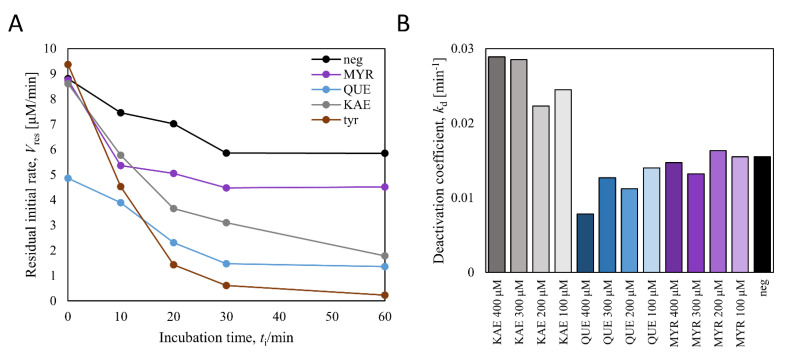
Deactivation kinetics of *Bm*Ty with 0.5 mM of kaempferol, quercetin, and myricetin. (**A**) and the deactivation coefficients with respect to 0.1~0.4 mM of putative deactivators (**B**). Each sample was represented with abbreviations, those are: neg: negative control (no putative deactivator), MYR: myricetin, QUE: quercetin, KAE: kaempferol, tyr: L-tyrosine.

To figure out the causes of such inhibition or deactivation in myricetin production, a preliminary test identifying deactivation kinetics of *Bm*Ty was performed (Figure 8). Briefly, flavonols of interest were incubated with *Bm*Ty in the similar condition of *ortho*-hydroxylation reactions and the residual monophenolase activity was analyzed by the dopachrome assay using L-tyrosine at different incubation points in time. The monophenolase activities decreased with an exponential decay profile with time, suggesting that the deactivation of *Bm*Ty appeared to follow 1^st^ order kinetics with deactivation coefficients (*k*_d_). However, the deactivation coefficients did not exhibit a positive linear correlation with regard to the concentration of incubated flavonols (Figure 8B). This represents that suicide deactivation of *Bm*Ty by flavonols did not happen. *Bm*Ty incubated with quercetin initially showed impaired activity compared with other cases (Figure 8A). 

**Figure 9:**
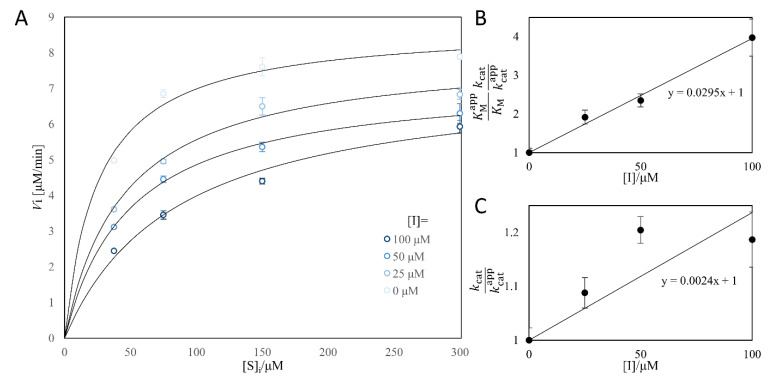
Mixed inhibition of *Bm*Ty by quercetin. Initial rates of *Bm*Ty were decreased when quercetin concentration was increased in the range of 0~100 μM quercetin and 37.5~300 μM L-tyrosine (**A**). Regression of two parameters derived two inversed inhibition constants, *K*_I1_ = 33.9 μM and *K*_I2_ = 417 μM, which means that quercetin is a highly competitive mixed inhibitor (**B**,**C**). Data were shown as averages and standard errors from triplicated samples.

As a result, to examine such possibility of inhibition by quercetin, Michaelis–Menten kinetics were evaluated by varying quercetin concentration (Figure 9A). Within the quercetin concentration range of 0~100 μM, somewhat mixed inhibition pattern was observed in monophenolase activity of *Bm*Ty with a highly competitive manner of *K*_I1_ = 33.9 μM and *K*_I2_ = 417 μM (Figure 9B,C). The values are similar to those of the Tyrosinase from *Agaricus bisphorous* [45]. Therefore, it appears that the accumulated quercetin from the monophenolase reaction of *Bm*Ty inhibited the *ortho*-hydroxylation activity of *Bm*Ty (i.e., monophenolase activity) prohibiting myricetin production.

## 4. Discussion

To date, tyrosinases were mainly used for medical applications, phenolic waste treatment and production of fine chemicals [4,5,38,49,50,51]. Our group has demonstrated that by incorporating borate chelation with a catechol intermediate generated by monophenolase activity of tyrosinase and using suitable reducing agents, several catecholic products could be successfully prepared with high yields. Outstanding examples are plant polyphenolic compounds including piceatannol and *ortho*-dihydroxy-isoflavones. This study demonstrated a rather simple approach of using bacterial tyrosinase for the synthesis of gallol products with commercial scale for the first time, since the enzyme is a coenzyme-free catalyst with high reaction rates unlike P450s. The gallol-producing bacterial tyrosinase, *Bm*Ty, was first isolated and characterized by the Fishman group [28]. According to their previous publications, *Bm*Ty showed broad substrate specificity for various monohydroxyphenols, dihydroxyphenols, and trihydroxyphenols, which was described with the crystal structures of the enzyme and a couple of mutants, and the broad substrate specificity has also been utilized for the preparation of functional catecholic compounds [4,52]. However, the previous reports on *Bm*Ty did not attempt to execute the production of gallols from monophenols or diphenols. Since catechols are susceptible to oxidation, it seems that acidic condition and additional reducing agents (i.e., L-ascorbate in this study) can prevent their autoxidation and over-oxidation allowing the enzyme to have a second chance of hydroxylation to intermediate diphenols, finally giving the accumulation of gallols.

Taking advantage of the newly developed biocatalysis, we could demonstrate to synthesize pharmaceutically valuable flavonoids, quercetin and myricetin using one tyrosinase, *Bm*Ty. Using kaempferol, product quercetin was obtained with 60% yield in one-hour reaction, and the titer was up to 1.2 mM from 2 mM of kaempferol. Notably, the achieved myricetin productivity was almost 10 times higher (i.e., 18 mg/L/h) than that of the previously reported one using a plant cytochrome P450s [21]. This synthesis platform has an excellent potential for various applications, since the facile in vitro syntheses shown in this paper were possible with high efficiency without NAD(P) coenzyme. However, the final myricetin concentration in the reaction solution was restricted (Figure 7). Such a drawback was caused by the product inhibition by the *ortho*-hydroxylation product quercetin. As quercetin was a competitive inhibitor of the mushroom tyrosinase [42,43,44,45], the contribution of competitive inhibition mode was higher than the uncompetitive mode when quercetin exerts mixed inhibition on *Bm*Ty. Escaping from competitive inhibition can be accomplished by increasing the dissociation constant of the inhibitor with mutational approaches in general cases. However, unfortunately, in this case, the inhibitor is another substrate for consecutive hydroxylation. One possible solution is increasing the turnover number (*k*_cat_, TON) of monophenolase activity. In this way, the *ortho*-hydrating reaction of *Bm*Ty will be rescued by fast conversion of quercetin without damaging catalytic efficiency(*k*_cat_/*K*_M_). However, it must be considered that the TON of diphenolase activity should remain constant or lower to prevent the unwanted over-oxidation of valuable products.

The monophenolase to diphenolase activity ratio is another big issue in tyrosinase reactions. In general, high monophenolase activity compared to diphenolase activity is favored for achieving a high yield of hydroxylated product, because diphenolase activity causes consumption of the desired *ortho*-hydroxylated product and generation of quinone product, leading to the formation of melanin analogues. Actually, many reports highlighted the importance of high monophenolase to diphenolase activity ratio for various tyrosinase mutants and natural isolates [53,54,55,56,57]. As results shown, monophenolase (L-tyrosine to L-dopachrome)-to-diphenolase activity (L-DOPA to L-dopachorme) ratio of *Bm*Ty increased with a decrease in reaction pH, (Figure 4) which is also a favored aspect for optimized mildly acidic reaction condition considering high stability of quercetin and myricetin in acidic conditions (Figure 2). Such an acidic environment might help to achieve the high productivity of myricetin, despite susceptibility to autoxidation of the gallol products.

The aforementioned problems may be further solved through enzyme engineering, applying selected mutations that modulate *k*_cat_ and *K*_M_ of individual monophenolase and diphenolase in *Bm*Ty homologues. A recent study reported that the monophenolase activity or diphenolase activity of type-III copper enzymes was controlled by the residues neighboring Cu-coordinating histidines such as HisB_1_ + 1 and HisB_2_ + 1 through modulating the basicity of histidine. Even monophenolase activity was generated by mutating aurone synthase, which did not have tyrosinase activity, and their mutational work showed plenty of mutants that vary *k*_cat_ and *K*_M_ of monophenolase or diphenolase activity, respectively.

Throughout considering flavonol stability and optimum enzyme-reaction pH, we have constructed an easy platform of catechol and gallol biosynthesis using tyrosinase without any coenzymes in mildly acidic conditions. Despite quercetin inhibition, this platform achieved 18 mg/L/h of myricetin productivity, currently the highest value ever published.

## 5. Conclusions

In this study, we found that pH stability was the crucial factor for the preparation of some readily oxidized flavonoids in the tyrosinase reaction. In the mild acidic condition, myricetin was facilely synthesized from kaempferol with the productivity of 18 mg/L/h in the tyrosinase one-pot reaction without any cofactors. Furthermore, some novel gallol flavonoids such as tricetin were successfully synthesized. We insist that this simple pH adjustment along with proper monophenolase activity of tyrosinase would be an efficient way to prepare versatile flavonoids hardly prepared by other ways.

## Figures and Tables

**Table 1:** Enzyme units of *Bm*Ty’s two activities at various pH conditions.

pH	Enzyme Unit [U/μmol Enzyme] ^1^	
	Monophenolase Activity	Diphenolase Activity
5	117 ± 6	202 ± 5
6	231 ± 19	494 ± 5
7	376 ± 19	699 ± 42
8	276 ± 23	879 ± 28

^1^ Values are shown as average ± standard deviation.

## Supplementary Materials

The following are available online at https://www.mdpi.com/article/10.3390/microorganisms9091866/s1, Table S1: Cloning information of the six bacterial tyrosinases and used primers., Table S2: GC-MS or LC-ESI-MS/MS anaylsis of hydroxylated flavanone and flavone products.

## Abbreviations

MRI	magnetic resonance imaging
CYP	cytochrome P450
NAD(P)	nicotinamide adenine dinucleotide (phosphate)
MS	mass spectrometry
HPLC	high performance liquid chromatography
Ni-NTA	nickel-nitrilotriacetic acid
UV/Vis	ultraviolet/visible light
GC	gas chromatography
ESI	electrospray ionization
EI	electron ionization
EA	ethyl acetate
KAE	kaempferol
QUE	quercetin
MYR	myricetin
CAT	catechol
QUI	*o*-quinone
L-DOPA	L-3,4-dihydroxyphenylalanine
LAA	L-ascorbic acid
PVP	poly(vinyl pyrrolidone)
BSTFA	*N*,*O*-Bis(trimethylsilyl)trifluoroacetamide
TMS	trimethylsilyl
TON	turnover number

## References

[1] McLarin M.-A., Leung I.K.H. (2020). Substrate specificity of polyphenol oxidase. Crit. Rev. Biochem. Mol. Biol..

[2] Solomon E.I., Sundaram U.M., Machonkin T.E. (1996). Multicopper Oxidases and Oxygenases. Chem. Rev..

[3] Muñoz-Muñoz J.L., Berna J., García-Molina M.d.M., Garcia-Molina F., Garcia-Ruiz P.A., Varon R., Rodriguez-Lopez J.N., Garcia-Canovas F. (2012). Hydroxylation of p-substituted phenols by tyrosinase: Further insight into the mechanism of tyrosinase activity. Biochem. Biophys. Res. Commun..

[4] Lee S.-H., Baek K., Lee J.-E., Kim B.-G. (2016). Using tyrosinase as a monophenol monooxygenase: A combined strategy for effective inhibition of melanin formation. Biotechnol. Bioeng..

[5] Lee N., Lee S.-H., Baek K., Kim B.-G. (2015). Heterologous expression of tyrosinase (MelC2) from Streptomyces avermitilis MA4680 in E. coli and its application for ortho-hydroxylation of resveratrol to produce piceatannol. Appl. Microbiol. Biotechnol..

[6] Min K., Park G.W., Yoo Y.J., Lee J.-S. (2019). A perspective on the biotechnological applications of the versatile tyrosinase. Bioresour. Technol..

[7] Ju K.-Y., Lee J.W., Im G.H., Lee S., Pyo J., Park S.B., Lee J.H., Lee J.-K. (2013). Bio-Inspired, Melanin-Like Nanoparticles as a Highly Efficient Contrast Agent for T1-Weighted Magnetic Resonance Imaging. Biomacromolecules.

[8] Wang W., Jing T., Xia X., Tang L., Huang Z., Liu F., Wang Z., Ran H., Li M., Xia J. (2019). Melanin-loaded biocompatible photosensitive nanoparticles for controlled drug release in combined photothermal-chemotherapy guided by photoacoustic/ultrasound dual-modality imaging. Biomater. Sci..

[9] Ju K.-Y., Kang J., Pyo J., Lim J., Chang J.H., Lee J.-K. (2016). pH-Induced aggregated melanin nanoparticles for photoacoustic signal amplification. Nanoscale.

[10] Kohri M., Nannichi Y., Taniguchi T., Kishikawa K. (2015). Biomimetic non-iridescent structural color materials from polydopamine black particles that mimic melanin granules. J. Mater. Chem. C.

[11] Barbehenn R.V., Constabel C.P. (2011). Tannins in plant–herbivore interactions. Phytochemistry.

[12] Mandal S.M., Chakraborty D., Dey S. (2010). Phenolic acids act as signaling molecules in plant-microbe symbioses. Plant Signal. Behav..

[13] Harborne J.B. (2013). The Flavonoids: Advances in Research Since 1980.

[14] Daino G.L., Frau A., Sanna C., Rigano D., Distinto S., Madau V., Esposito F., Fanunza E., Bianco G., Taglialatela-Scafati O. (2018). Identification of Myricetin as an Ebola Virus VP35–Double-Stranded RNA Interaction Inhibitor through a Novel Fluorescence-Based Assay. Biochemistry.

[15] Bimakr M., Rahman R.A., Taip F.S., Ganjloo A., Salleh L.M., Selamat J., Hamid A., Zaidul I.S.M. (2011). Comparison of different extraction methods for the extraction of major bioactive flavonoid compounds from spearmint (*Mentha spicata* L.) leaves. Food Bioprod. Process..

[16] Azmir J., Zaidul I.S.M., Rahman M.M., Sharif K.M., Mohamed A., Sahena F., Jahurul M.H.A., Ghafoor K., Norulaini N.A.N., Omar A.K.M. (2013). Techniques for extraction of bioactive compounds from plant materials: A review. J. Food Eng..

[17] Chaves J.O., de Souza M.C., Da Silva L.C., Lachos-Perez D., Torres-Mayanga P.C., Machado A., Forster-Carneiro T., Vázquez-Espinosa M., González-De-Peredo A.V., Barbero G.F. (2020). Extraction of Flavonoids From Natural Sources Using Modern Techniques. Front. Chem..

[18] Azwanida N.N. (2015). A Review on the Extraction Methods Use in Medicinal Plants, Principle, Strength and Limitation. Med. Aromat. Plants.

[19] Wang L., Weller C.L. (2006). Recent advances in extraction of nutraceuticals from plants. Trends Food Sci. Technol..

[20] Pandey R.P., Parajuli P., Koffas M.A., Sohng J.K. (2016). Microbial production of natural and non-natural flavonoids: Pathway engineering, directed evolution and systems/synthetic biology. Biotechnol. Adv..

[21] Du Y., Yang B., Yi Z., Hu L., Li M. (2020). Engineering Saccharomyces cerevisiae Coculture Platform for the Production of Flavonoids. J. Agric. Food Chem..

[22] Marín L., Gutiérrez-Del-Río I., Entrialgo-Cadierno R., Villar C.J., Lombó F. (2018). De novo biosynthesis of myricetin, kaempferol and quercetin in Streptomyces albus and Streptomyces coelicolor. PLoS ONE.

[23] Jones J.A., Vernacchio V.R., Sinkoe A.L., Collins S.M., Ibrahim M.H.A., Lachance D.M., Hahn J., Koffas M.A.G. (2016). Experimental and computational optimization of an Escherichia coli co-culture for the efficient production of flavonoids. Metab. Eng..

[24] Deze Kong S.L., Smolke C.D. (2020). Discovery of a previously unknown biosynthetic capacity of naringenin chalcone synthase by heterologous expression of a tomato gene cluster in yeast. Sci. Adv..

[25] Ayabe S.-I., Akashi T. (2006). Cytochrome P450s in flavonoid metabolism. Phytochem. Rev..

[26] Lee P.-G., Lee S.-H., Kim J., Kim E.-J., Choi K.-Y., Kim B.-G. (2018). Polymeric solvent engineering for gram/liter scale production of a water-insoluble isoflavone derivative, (S)-equol. Appl. Microbiol. Biotechnol..

[27] Lee P.-G., Lee S.-H., Hong E.Y., Lutz S., Kim B.-G. (2019). Circular permutation of a bacterial tyrosinase enables efficient polyphenol-specific oxidation and quantitative preparation of orobol. Biotechnol. Bioeng..

[28] Shuster V., Fishman A. (2009). Isolation, Cloning and Characterization of a Tyrosinase with Improved Activity in Organic Solvents from Bacillus megaterium. J. Mol. Microbiol. Biotechnol..

[29] Moon Y.J., Wang L., DiCenzo R., Morris M.E. (2008). Quercetin pharmacokinetics in humans. Biopharm. Drug Dispos..

[30] Wang W., Sun C., Mao L., Ma P., Liu F., Yang J., Gao Y. (2016). The biological activities, chemical stability, metabolism and delivery systems of quercetin: A review. Trends Food Sci. Technol..

[31] Xiang D., Wang C.G., Wang W.-Q., Shi C.-Y., Xiong W., Wang M.-D., Fang J.-G. (2017). Gastrointestinal stability of dihydromyricetin, myricetin, and myricitrin: An in vitro investigation. Int. J. Food Sci. Nutr..

[32] Yao Y., Lin G., Xie Y., Ma P., Li G., Meng Q., Wu T. (2014). Preformulation studies of myricetin: A natural antioxidant flavonoid. Die Pharmazie Int. J. Pharm. Sci..

[33] Franklin S.J., Myrdal P.B. (2015). Solid-State and Solution Characterization of Myricetin. AAPS PharmSciTech.

[34] Jang J.-H., Park Y.-D., Ahn H.-K., Kim S.-J., Lee J.-Y., Kim E.-C., Chang Y.-S., Song Y.-J., Kwon H.-J. (2014). Analysis of Green Tea Compounds and Their Stability in Dentifrices of Different pH Levels. Chem. Pharm. Bull..

[35] Schüsler-Van Hees M.T.I.W., Beijersbergen Van Henegouwen G.M.J., Stoutenberg P. (1985). Autoxidation of catechol(amine)s. Pharmaceutisch Weekblad.

[36] Gao R., Yuan Z., Zhao Z., Gao X. (1998). Mechanism of pyrogallol autoxidation and determination of superoxide dismutase enzyme activity. Bioelectrochem. Bioenerg..

[37] Lee N., Kim E.J., Kim B.-G. (2012). Regioselective Hydroxylation of trans-Resveratrol via Inhibition of Tyrosinase from Streptomyces avermitilis MA4680. ACS Chem. Biol..

[38] Kim S.-H., Lee S.-H., Lee J.-E., Park S.J., Kim K., Kim I.S., Lee Y.-S., Hwang N.S., Kim B.-G. (2018). Tissue adhesive, rapid forming, and sprayable ECM hydrogel via recombinant tyrosinase crosslinking. Biomaterials.

[39] Seo J.-H., Park J., Kim E.-M., Kim J., Joo K., Lee J., Kim B.-G. (2014). Subgrouping Automata: Automatic sequence subgrouping using phylogenetic tree-based optimum subgrouping algorithm. Comput. Biol. Chem..

[40] Son H.F., Lee S.-H., Lee S.H., Kim H., Hong H., Lee U.-J., Lee P.-G., Kim B.-G., Kim K.-J. (2018). Structural Basis for Highly Efficient Production of Catechol Derivatives at Acidic pH by Tyrosinase from Burkholderia thailandensis. ACS Catal..

[41] Chiang C.-M., Wang D.-S., Chang T.-S. (2016). Improving Free Radical Scavenging Activity of Soy Isoflavone Glycosides Daidzin and Genistin by 3′-Hydroxylation Using Recombinant *Escherichia coli*. Molecules.

[42] Li Y., Zafar A., Kilmartin P.A., Reynisson J., Leung I.K.H. (2017). Development and Application of an NMR-Based Assay for Polyphenol Oxidases. ChemistrySelect.

[43] Kim D., Park J., Kim J., Han C., Yoon J., Kim N., Seo J., Lee C. (2006). Flavonoids as Mushroom Tyrosinase Inhibitors: A Fluorescence Quenching Study. J. Agric. Food Chem..

[44] Kubo I., Kinst-Hori I., Chaudhuri S.K., Kubo Y., Sánchez Y., Ogura T. (2000). Flavonols from Heterotheca inuloides : Tyrosinase Inhibitory Activity and Structural Criteria. Bioorg. Med. Chem..

[45] Kubo I., Kinst-Hori I. (1999). Flavonols from Saffron Flower: Tyrosinase Inhibitory Activity and Inhibition Mechanism. J. Agric. Food Chem..

[46] Fan M., Zhang G., Hu X., Xu X., Gong D. (2017). Quercetin as a tyrosinase inhibitor: Inhibitory activity, conformational change and mechanism. Food Res. Int..

[47] Garcia-Jimenez A., Teruel-Puche J.A., Garcia-Ruiz P.A., Saura-Sanmartin A., Berna J., Rodríguez-López J.N., Garcia-Canovas F. (2018). Action of tyrosinase on caffeic acid and its n-nonyl ester. Catalysis and suicide inactivation. Int. J. Biol. Macromol..

[48] Land E.J., Ramsden C.A., Riley P.A. (2007). The mechanism of suicide-inactivation of tyrosinase: A substrate structure investigation. Tohoku J. Exp. Med..

[49] Munoz-Munoz J.L., Berná J., Garcia-Molina F., García-Ruiz P.A., Tudela J., Rodriguez-Lopez J.N., García-Cánovas F. (2012). Unravelling the suicide inactivation of tyrosinase: A discrimination between mechanisms. J. Mol. Catal. B Enzym..

[50] Kim S.-H., An Y.-H., Kim H.D., Kim K., Lee S.-H., Yim H.-G., Kim B.-G., Hwang N.S. (2018). Enzyme-mediated tissue adhesive hydrogels for meniscus repair. Int. J. Biol. Macromol..

[51] Chávez-Béjar M.I., Balderas-Hernandez V.E., Gutiérrez-Alejandre A., Martínez A., Bolivar F., Gosset G. (2013). Metabolic engineering of Escherichia coli to optimize melanin synthesis from glucose. Microb. Cell Factories.

[52] Wada S., Ichikawa H., Tastsumi K. (1995). Removal of phenols and aromatic amines from wastewater by a combination treatment with tyrosinase and a coagulant. Biotechnol. Bioeng..

[53] Kampatsikas I., Bijelic A., Pretzler M., Rompel A. (2017). Three recombinantly expressed apple tyrosinases suggest the amino acids responsible for mono- versus diphenolase activity in plant polyphenol oxidases. Sci. Rep..

[54] Goldfeder M., Kanteev M., Adir N., Fishman A. (2013). Influencing the monophenolase/diphenolase activity ratio in tyrosinase. Biochim. Biophys. Acta (BBA) Proteins Proteom..

[55] Do H., Kang E., Yang B., Cha H.J., Choi Y.S. (2017). A tyrosinase, mTyr-CNK, that is functionally available as a monophenol monooxygenase. Sci. Rep..

[56] Hernandez-Romero D., Sanchez-Amat A., Solano F. (2006). A tyrosinase with an abnormally high tyrosine hydroxylase/dopa oxidase ratio. FEBS J..

[57] Solem E., Tuczek F., Decker H. (2016). Tyrosinase versus Catechol Oxidase: One Asparagine Makes the Difference. Angew. Chem. Int. Ed. Engl..

