# Supplementary material for: Polyphenol-Hydroxylating Tyrosinase Activity under Acidic pH Enables Efficient Synthesis of Plant Catechols and Gallols
Source: Microorganisms. 2021 Sep 2;9(9):1866. doi: 10.3390/microorganisms9091866 (PMC8466947; doi:10.3390/microorganisms9091866)
Supplement: Supplementary file 1 [file microorganisms-09-01866-s001.zip › microorganisms-1304450-supplementary.pdf]

Supporting information

Polyphenol-hydroxylating tyrosinase activity under acidic pH enables efficient synthesis of plant catechols and gallols

Hanbit Song[1,2*], Pyung-Gang Lee[1,2,3*], Hyun Kim[1,2], Uk-Jae Lee[1,2,] Sang-Hyuk Lee[1,2], Joonwon Kim[1,2], Byung-Gee Kim[1,2,4,5†]

[1]School of Chemical and Biological Engineering, Seoul National University, Seoul, 08826, South Korea

[2]Institute of Molecular Biology and Genetics, Seoul National University, Seoul, 08826, South Korea

[3]Institute of Engineering Research, Seoul National University, Seoul, 08826, South Korea

[4]Bio-MAX/N-Bio Institute, Seoul National University, Seoul, 08826, South Korea

[5]Institute for Sustainable Development(ISD), Seoul National University, Seoul, 08826, South Korea

*Hanbit Song and Pyung-Gang Lee contributed equally to this work.

†Corresponding author

Table S1. Cloning information of the six bacterial tyrosinases and used primers.

Cloned tyrosinase	Origin of tyrosinase gene	Vector	Cloning method	Restriction enzyme	Primer sequences (5’ to 3’)
*Bm*Ty	*Bacilius megaterium* ATCC 10778	pET28a (Kan^R)	already cloned[1]	N/A	N/A
*Bt*Ty	*Burkholderia thailandensis* E264		already cloned[2]	N/A	N/A
*Bc*Ty	*Burkholderia cepacia* KCCM 41422 (ATCC 25416)		circular polymerase extension cloning[3]	N/A	for insert amplification:<br>GGAGATATACCATGGCAAATAACGCATCTGGAGTCAG<br>CGGCCGCAAGCTTTCGGACCTCGAGCCGGA<br>for vector amplification:<br>AGATGCGTTATTTGCCATGGTATATCTCC<br>GTCCGAAAGCTTGCGGCCG
*Kg*Ty	*Kitasatospora griseola* MF730-N6		restriction enzyme digestion and ligation	NcoI (forward)	TAT**CCATGG**CCACGACCCCAGGAAACCC
				HindIII (reverse)	TAT**AAGCTT**CGCGTCCTGCACCAGCAG
*Pg*Ty	*Photobacterium galatheae* S2753		restriction enzyme digestion and ligation	BamHI (forward)	ATA**GGATCC**ACGACTGTCGCGAAACTC
				XhoI (reverse)	ATA**CTCGAG**CTGTTTTGCAAAAGTTCGC
*Sa*Ty	*Streptomyces avermitilis* MA4680	pETDuet (Amp^R)	already cloned[1]	N/A	N/A

Table S2. GC-MS or LC-ESI-MS/MS anaylsis of hydroxylated flavanone and flavone products.

Reaction products	Employed MS type	Structural formula	Retention time [min]	m/z of $[M+H^+]$ (% relative abundance[b])	m/z of fragments (% relative abundance)
eriodictyol[a]	GC-MS	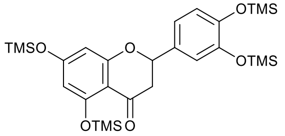	15.78	577 (14)	562 (100), 560 (9), 549 (5), 505 (12), 474 (12), 383 (5), 370 (21), 324 (6), 307 (19), 298 (9), 280 (15), 267 (78), 265 (5), 179 (18), 73 (38)
3',5'-dihydroxy-naringenin[a]	GC-MS	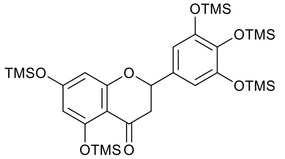	16.24	665 (20)	650 (63), 648 (5), 593 (13), 576 (5), 562 (13), 370 (24), 356 (100), 324 (5), 307 (34), 298 (8), 282 (4), 267 (23), 265 (20), 237 (8), 235 (5), 207 (5), 193 (4), 147 (5), 133 (8), 73 (21)
luteolin	LC-ESI-MS/MS	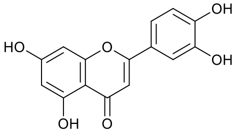	13.16	287 (100)	269 (8), 241 (15), 213 (6), 179 (7), 161 (9), 153 (94), 137 (13), 135 (41), 117 (10), 89 (14), 67 (6)
tricetin	LC-ESI-MS/MS	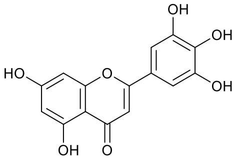	8.75	303 (100)	285 (37), 257 (47), 229 (30), 215 (2), 203 (35), 177 (6), 153 (22), 151 (15), 145 (1), 105 (2), 77 (13)

a: Data are according to TMSylated products. b: For LC-ESI-MS/MS analysis, % relative abundance of parental ion m/z is set 100%.

References

1 Lee, S. H., Baek, K., Lee, J. E. & Kim, B. G. Using tyrosinase as a monophenol monooxygenase: A combined strategy for effective inhibition of melanin formation. *Biotechnology and bioengineering* **113**, 735-743 (2016).

2 Son, H. F. *et al.* Structural Basis for Highly Efficient Production of Catechol Derivatives at Acidic pH by Tyrosinase from Burkholderia thailandensis. *ACS Catalysis* **8**, 10375-10382 (2018).

3 Quan, J. & Tian, J. Circular polymerase extension cloning for high-throughput cloning of complex and combinatorial DNA libraries. *Nat Protoc* **6**, 242-251, doi:10.1038/nprot.2010.181 (2011).
